# Supporting Healthcare Workers during COVID-19 Pandemic and beyond

**DOI:** 10.31729/jnma.5605

**Published:** 2021-10-31

**Authors:** Badri Man Shrestha

**Affiliations:** 1 Sheffield Kidney Institute, Sheffield Teaching Hospitals National Health Service Trust, Sheffield, United Kingdom

Severe acute respiratory syndrome coronavirus-2 (SARS-CoV-2) continues to spread globally, with more than 220 million cases of Coronavirus disease 2019 (COVID-19) and more than four million deaths as of 6 September, 2021.^1^ Healthcare workers (HCWs), which include doctors, nurses, laboratory technicians, pharmacists, information technology and ambulance staffs and many other staffs directly or indirectly involved in patient management, have been integral to the response to COVID-19. The dedication and sacrifices made by the HCWs during the COVID-19 pandemic is well recognised globally. The nature of their job puts them at increased risk of contracting SARS-CoV-2 and hence subsequently transmitting it to their household, workplace contacts, or both, which has been a cause of concern and has impacted negatively on their mental health. This editorial aims to highlight the risks of COVID-19 to the HCWs, effects on their mental health and strategies to be adopted to address these issues.

A prospective, observational cohort study in the UK and the USA of the general community (n = 20,35,395), including front-line HCWs (n = 99,795), showed the front-line HCWs were at elevenfold increased risk for reporting a positive COVID-19 test compared with the general community. Adequacy of personal protective equipment (PPE), clinical setting and ethnic background, particularly those from Black, Asian, and minority ethnic (BAME) backgrounds were important factors for the increased risk.^2^

A study published from Scotland, involving a cohort of 158445 HCWs (57% being patient facing) and 2,29,905 household members, showed a threefold increased risk of hospital admission due to COVID-19 among patients facing HCWs compared to non-patient facing HCWs. The household members of patients facing HCWs had a twofold increased risk of hospital admission due to COVID-19 compared to non-patient facing HCWs. However, the risk of hospital admission due to COVID-19 in non-patient facing healthcare workers and their households was similar to the risk in the general population.^3^

The COVID-19 pandemic has overstretched healthcare systems globally including the resourced countries, where the HCWs are struggling with long working hours, fatigue and extreme psychological stress from the fear of contracting the virus and infecting their family members, grief from seeing so many patients die and anger over healthcare disparities and system failures. Frontline HCWs, particularly working in the intensive care units, respiratory and emergency medicine, and ambulance services, are exposed to patients infected with the coronavirus. During lockdowns, the most essential workers are unable to protect themselves by working from home. Insufficient physical distancing is a major contributor to the work-related COVID-19 outbreak. Furthermore, HCWs are affected by wider societal and economic tensions, including the impacts of social distancing and fewer social resources. This complex combination of stressors can lead to anxiety, depression, adjustment disorders, accelerated burnout and post-traumatic stress disorders, which may be implicated in suicides.^4,5^

The ethos of the medical profession, deeply entrenched and counterproductive views, and expectations from the HCWs, can be barriers to bolstering emotional wellbeing. The culture of medicine has always reinforced the belief that self-care is selfish and physical and emotional exhaustion is part of the job. They are expected to sacrifice personally and be resilient. Doctors, for instance, have a duty to care for patients as well as a duty to care for their own families by protecting them and hence themselves from infection.^6^ Self-referral for mental health problems has always been an issue because of its association with stigmatisation, resulting in isolation, suffering in silence and protracted recovery. Historically, medical institutions have not looked upon HCWs suffering from mental health issues with compassion, which has eroded the trust of the HCWs in their organisation. It is recognised that emotional stressors are often occupational hazards rather than mental health problems.^7^

It is, therefore, important to recognise the contributions made by the HCWs during the COVID-19 pandemic despite all adversities and optimise the working environment to preserve their morale and well-being. The well-being of the HCWs should be an organizational priority, support programs should be adequately resourced to mitigate the workplace stressors and leaders should be held accountable for outcomes related to well-being. This can be achieved by ensuring improvement in workplace efficiency, supporting appropriate nutrition, rest and sleep periods, increasing supply of PPE, regular testing for the virus, assessment of the risk of individual staff using appropriate tools, government support to the family members in the event of unemployment and strengthening the regular communication with organisational leaders.^8,9^ An institutional approach comprising of an employee assisting programme offering counselling and debriefing is essential. Maintenance of registry with prospective records of short- and long-term outcomes of COVID-19 among HCWs and their families will help further evaluation of the risks and develop strategies to support the HCWs during the pandemic and beyond.^10^
